# Effect of super absorbent hydrogel on hydro-physical properties of soil under deficit irrigation

**DOI:** 10.1038/s41598-024-57786-5

**Published:** 2024-04-01

**Authors:** Rasha Abdelghafar, Ahmed Abdelfattah, Harby Mostafa

**Affiliations:** Department of Agricultural and Biosystems Engineering, Faculty of Agriculture, Benha University, Moshtohor, Toukh, Kalubia Egypt

**Keywords:** Super absorbent materials, Water stress, Soil hydro-physical properties, Water use efficiency, Plant ecology, Plant stress responses

## Abstract

Due to water scarcity challenges, efficient management of irrigation water is becoming crucial. Water use efficiency (WUE) involves increasing crop productivity without increasing water consumption. This study was carried out to study the effect of hydrogel, deficit irrigation and soil type on WUE, soil hydro-physical properties and lettuce productivity. For this purpose, four irrigation treatments (100%, 85%, 70% and 60% of full irrigation requirements), four hydrogel concentrations (0, 0.1, 0.2 and 0.3% w/w) and three soil textural classes (clay, loamy sand, and sandy-clay soil) were conducted in pot experiment at open field during two consecutive seasons. The results revealed that crop growth parameters and soil hydro-physical properties were significantly affected by hydrogel application rates. Hydrogel addition significantly enhanced head fresh and dry weights, chlorophyll content, number of leaves and WUE. Application of hydrogel at 0.3% and 85% of irrigation requirements achieved the highest WUE without significant yield reductions. Changes in the studied hydro-physical properties of soil were more dependent on soil texture and hydrogel application rate than on the amount of irrigation water. The significant decrease in soil saturated hydraulic conductivity and bulk density confirms that super absorbent hydrogels could be recommended to improve soil water retention and enhance water use efficiency under deficit irrigation conditions.

## Introduction

Egypt is facing significant water scarcity challenges. The country is located in a mostly arid region and is characterized by hot dry climate. The average annual precipitation in Egypt ranges from less than 25 mm in some parts of the Western Desert to around 200 mm in the northern coastal regions. Due to the limited rainfall, Egypt depends mainly on the Nile River for its water resources^1–3^. The Nile provides approximately 90% of Egypt’s freshwater supply, and the agricultural sector consumes about 85% of the country’s total water consumption for irrigation purpose. The rapid increase in population and the increasing demand for water have necessitated the adoption of efficient water-saving strategies in irrigated agriculture^4,5^.

The concept of water use efficiency (WUE) involves the challenge of how to increase crop productivity without increasing water consumption. Enhancing WUE ensures maximizing yield per unit of water, rather than yield per unit of land. Therefore, improving WUE will be reflected in sustainable use of limited water resources^6,7^. Deficit irrigation (DI) and the incorporation of soil amendments, including hydrogels, have been proposed to enhance WUE and mitigate negative impacts of water stress in arid and semi-arid regions^8,9^.

Deficit irrigation (DI) is defined as an irrigation management strategy, in which plants are intentionally exposed to a certain level of water stress throughout the entire growing season or at certain growth stages. DI implies that water is supplied at levels below crop water requirements or full evapotranspiration (ETc)^10,11^. Although yields will be reduced under deficit irrigation, the reduction in irrigation costs and water conservation may compensate for yield reductions. When the amount of land under irrigation is constrained by limited water availability, the economic returns to water will be maximized by reducing the depth of water applied and increasing the area of land under irrigation^12^. In order to ensure the success of DI irrigation, it is necessary to consider the ability of the soil to retain water. Sandy soil is characterized by excessive infiltration rate, low fertility and low water and nutrient holding capacity, therefore DI in sandy soils may expose plants to severe water stress. Therefore, the success of DI in these soils is more likely with modification in soil hydro-physical characteristics^13,14^.

Super absorbent hydrogels may offer a promising solution for improving water retention and hydro-physical characteristics of sandy soils. Super absorbent hydrogels are substances that can absorb and retain large amounts of water, about 400–500 times its own weight^15,16^. They have been used in agriculture as a soil amendment to improve water management and increase crop yields. According to^17,18^, the addition of hydrogel increased the water holding capacity of sandy soils and reduced deep percolation losses.

One of the main benefits of hydrogels in agriculture is their ability to retain water and release it slowly over time, which can help plants survive drought conditions. Hydrogels can also reduce water consumption and increase WUE by reducing both runoff and deep percolation. In addition to water management, hydrogels can also improve soil structure and nutrient availability. They can help loosen compacted soil, increase aeration, and improve the soil’s ability to hold nutrients. This can lead to healthier plants with better root development and increased yields^19^.

Vegetable crops production such as lettuce, is considered a major challenge in terms of efficient irrigation management, due to the crop’s sensitivity to water stress. Several studies have indicated that vegetable crops are very sensitive to water deficits. Water stress in vegetable crops is often accompanied by significant yield reductions and poor marketing quality^12,20^. The sensitivity of vegetable crops, especially fast-growing ones, to water stress may be attributed to two main reasons. First, the shallow root system of most vegetable crops restricts their ability to absorb water from deeper soil layers under water deficit conditions^21,22^. Secondly, marketing of vegetable crops depends on their signs of freshness, which is a direct result of their water content and deteriorates significantly when exposed to water stress^23,24^. From this perspective, incorporation of hydrogels with DI as drought-resistant strategy can sustain vegetable productivity and quality and save water in water-limited arid and semi-arid regions^25,26^.

Previous studies have concluded that hydrogels improve soil physical properties while also increasing WUE and growth parameters in arid and semi-arid areas. However, information concerning the effectiveness of hydrogel in reducing the adverse effect of water stress on leafy vegetables is still limited. Moreover, more research is needed to determine the optimal application rates of hydrogels for different crops and soil types. The aim of this study is to study the effect of super absorbent hydrogel application rate on soil hydro-physical properties and lettuce plant growth parameters, under different soils and irrigation levels.

## Materials and methods

### Experimental site

The study was carried out in pot experiment during two consecutive seasons 2022 and 2023 in Nagrig, Basyoun city, El-Gharbia governorate, Egypt (30° 58′ N, 30° 52′ E). Soils in the pots were collected from the surrounding farmlands in El-Gharbia governorate, from the top 20 cm. The collected soils were air-dried and sieved through a 2 mm sieve to remove any organic residues or visible roots and then homogenized by mixing thoroughly before use. The chemical and physical properties of the soils are shown in Tables 1 and 2. The study area is characterized by an arid climate, the mean annual precipitation and reference evapotranspiration are 50.3 mm and 1670 mm, respectively. The average monthly values of weather conditions in the experimental site are shown in (Table 3).

**Table 1:** Chemical properties of the experimental soils.

	pH	E.C (dS/m)	Soluble cations (ppm)				Soluble anions (ppm)			
			Ca^++^	K^+^	Na^+^	Mg^++^	Cl^−^	SO4^=^	HCO3^−^	CO3^=^
S_1_	7.75	1.32	2.31	0.88	3.54	2.64	1.65	4.84	2.86	–
S_2_	7.43	0.27	0.64	0.43	1.40	0.51	0.91	0.56	1.49	–
S_3_	7.52	0.32	0.8	0.41	1.20	0.50	1.4	1.21	0.30	–

**Table 2:** Physical and hydro-physical soil properties of the tested soils.

Soil	Particle size distribution (%)			Texture class	Hydro-physical properties				
	Sand	Silt	Clay		FC (%)	P.W.P (%)	AW (%)	K (cm h^−1^)	BD (g cm^−3^)
S_1_	12.00	28.00	60.00	Clay	41.40	20.00	21.40	0.45	1.20
S_2_	81.00	8.45	10.55	Loamy sand	18.29	9.11	9.18	2.65	1.58
S_3_	46.00	15.00	39.00	Sandy clay	27.80	14.7	13.10	1.22	1.33

*FC* field capacity, *P.W.P* permanent wilting point, *AW* available water, *K* Hydraulic conductivity, *BD* bulk density.

**Table 3:** Average of monthly meteorological data during 2022 and 2023 in the experimental site.

Month	T		RH		Ws	Rn	ETo	P
	Min	Max	Min	Max				
March	8.8	24.1	45	84	2.9	18.8	4.05	5.5
April	12.3	28.0	47.4	81	2.7	22.5	5.14	3
May	16.0	33.2	44.7	78	2.8	25.5	6.56	2

*T* temperature (°C), *RH* relative humidity (%), *Ws* wind speed (m/sec), *Rn* solar radiation (MJ/m^2^/day), *ETo* reference evapotranspiration (mm), *P* effective rainfall (mm).

### Experimental design

To study the effect of different levels of super-absorbent hydrogel on soil hydro-physical properties under deficit irrigation, a factorial experiment was carried out. The experimental treatments included (1) irrigation amount factor at four levels: 100 (I_1_), 85 (I_2_), 70 (I_3_) and 60% (I_4_) of ETc, (2) soil type factor at three levels: clay (S_1_), loamy sand (S_2_), and sandy-clay soil (S_3_) and (3) four hydrogel concentrations: 0 (H_0_), 0.1(H_1_), 0.2 (H_2_) and 0.3% (H_3_) (w/w). Treatments were arranged in split plot experimental layout in a Randomized Complete Blocks Design (RCBD) with three replicates for each treatment. Soil type was arranged in the main plots and hydrogel levels, irrigation amounts and their combinations, were considered as sub-plots. The experiment is composed of three replications for each treatment and each replication is made of one pot; in total 144 pots were used.

### Transplantation and irrigation management

Lettuce seedlings were obtained from commercial nursery and transplanted in plastic pots (35 cm inner diameter, and 30 cm height). Each pot was filled with 12 kg of air-dried soil and placed outdoors in open field conditions. Two seedlings were transplanted in each pot on the 1st of March of each growing season. Pots were irrigated with tap water to field capacity, chemical analysis of irrigation water is shown in Table 4. Plants were thinned to one plant per pot 25 days after transplanting. Nitrogen (N), phosphate (P), potassium (K) and microelements were uniformly applied to all pots according to the recommendations of Egyptian ministry of Agriculture.

**Table 4:** Chemical analysis of irrigation water.

PH	EC dS/m	EC ppm	Soluble cations (ppm)				Soluble anions (ppm)			
			Ca^++^	Mg^++^	K^+^	Na^+^	HCO_3_^−^	CO_3_^−^	SO_4_^−^	Cl^−^
7.13	0.5	320	2.18	1.12	0.35	1.14	2.64	–	0.79	1.35

The irrigation volumes were determined by measuring volumetric soil water content using the VH400 dielectric soil moisture sensor probe (Vegetronix, Inc., Riverton, Utah, USA). The output of the VH400 sensors was displayed using the VG-METER-200 handheld moisture meter (Vegetronix, Inc., Riverton, Utah, USA). Irrigation events were scheduled when soil moisture was depleted below field capacity in the top 10 cm of soil due to shallow root depth of lettuce (5–10 cm). The amount of moisture depletion was calculated before each irrigation and summed to obtain the seasonal water consumption.

### Plant measured parameters

The harvest took place when the plants reached the marketable size (67–70 days after transplanting in pots). Chlorophyll content was measured immediately before harvest using SPAD-502 portable nondestructive chlorophyll meter (Konica Minolta Inc., Tokyo, Japan). Chlorophyll measurement was performed on a single fully developed leaf in the middle of lettuce head. After harvesting, plant vegetative growth parameters (e. g., fresh and dry weight of aerial part, root wet and dry weight, and number of leaves) were measured and recorded. Dry weights were determined after oven drying at 70 °C until constant weight is reached.

Water use efficiency (WUE) (kg m^−3^) indicates the biomass produced per unit of water used by the crop. It was evaluated as the ratio between the marketable yield (kg ha^−1^) and seasonal crop evapotranspiration (mm).

### Soil hydro-physical properties

The impact of different concentrations of hydrogel on hydro-physical characteristics of different soils was evaluated. The evaluation was based on studying the change in both the hydraulic conductivity coefficient and the bulk density of the soil before and after the experiment using undisturbed soil samples^27^.

Saturated hydraulic conductivity K_s_ (cm h^−1^) was calculated using Darcy’s law as follow:

$$\frac{Q}{A} = K_{s} \frac{dh}{{dl}}$$

where Q: water flow rate passing through soil column (cm^3^ s^−1^), A: area of soil column (cm^2^), dh/dl: hydraulic gradient. Soil bulk density $\rho$ (g cm^−3^) was determined using the following equation:

$$\rho = \frac{{M_{d} }}{{V_{t} }}$$

where ${M}_{d}$: mass of dry soil (g) , ${V}_{t}$: total or bulk volume of soil (cm^3^).

### Statistical analysis

Analysis of variance of the obtained data in 2022 and 2023 seasons was carried out using Statistix 10 statistical software. For means comparisons, least significant difference (LSD) was applied (*P* < 0.05 significance level).

### Ethical approval

We confirm that all methods were carried out in accordance with relevant guidelines in the method section.

## Results

### Effect of different treatments on lettuce growth parameters

**Figure 1:**
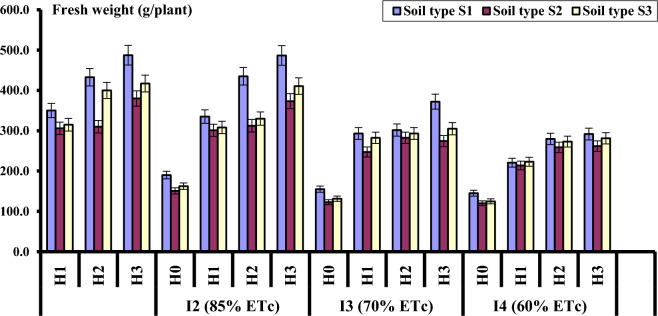
Head fresh weight as affected by irrigation level, hydrogel rate and soil type.

Data in Fig. 1 and Table 5 shows the effect of different treatments (irrigation levels, hydrogel application rates and soil types) on head fresh weight (g/plant). The average values for both growing seasons revealed that irrigation levels significantly affected lettuce fresh weight (*P* ˂ 0.05%). The highest values were achieved by full irrigation I_1_ (100% ET_C_), compared to I_4_ (DI with 60% ET_C_), which gave the lowest fresh weight values.

**Table 5:** Effect of irrigation level, hydrogel rate and soil type on head fresh weight of lettuce (g/plant).

Irrigation level (A)	Hydrogel levels (B)	Soil type (C)			Mean
		S_1_	S_2_	S_3_	(A × B)
I_1_ (FI)	H0 (Control)	210.0^s^	155.0^w^	170.0^u^	**178.3**^**K**^
	H1 (0.1%)	350.0^g^	306.0^jk^	315.0^i^	**323.7**^**E**^
	H2 (0.2%)	432.7^b^	310.0^ij^	400.0^d^	**380.9**^**C**^
	H3 (0.3%)	487.3^a^	380.0^e^	417.0^c^	**428.1**^**A**^
Mean (A × C)		**370.0**^**A**^	**287.8**^E^	**325.5**^C^	**327.8**^A^
I_2_ (85%ETc)	H0 (Control)	190.0^t^	150.7^wx^	162.3^v^	**167.7**^**L**^
	H1 (0.1%)	335.0^h^	301.0^k^	308.0^i−k^	**314.7**^**F**^
	H2 (0.2%)	435.0^b^	312.3^ij^	330.0^h^	**359.1**^**D**^
	H3 (0.3%)	486.7^a^	373.7f.	410.7^c^	**423.6**^**B**^
Mean (A × C)		**361.7**^**B**^	**284.3**^F^	**302.0**^D^	**316.3**^B^
I_3_ (70%ETc)	H0 (Control)	155.0^w^	122.7^z^	131.3^y^	**136.3**^**M**^
	H1 (0.1%)	293.0 l	247.3^q^	282.3^m^	**274.2**^**I**^
	H2 (0.2%)	301.7^k^	282.3^m^	293.0l	**292.1**^**J**^
	H3 (0.3%)	372.0^f^	274.3^no^	305.0^jk^	**317.1**^**I**^
Mean (A × C)		**280.4**^**G**^	**231.7**^I^	**252.9**^H^	**255.0**^C^
I_4_ (60%ETc)	H0 (Control)	145.0^x^	120.0^z^	124.7^z^	**129.9**^**N**^
	H1 (0.1%)	220.7^r^	213.7^s^	223.0^r^	**219.1**^**J**^
	H2 (0.2%)	279.7^m–o^	258.7^p^	273.0^o^	**270.5**^**I**^
	H3 (0.3%)	291.7 l	261.7^p^	281.2^mn^	**278.2**^**H**^
Mean (A × C)		**234.3**^**I**^	**213.5**^K^	**225.5**^J^	**224.4**^D^
Mean (B × C)		**175.0**^**I**^	**137.1**^K^	**147.1**^J^	**153.1**^D^
		**299.7**^**E**^	**267.0**^H^	**282.1**^G^	**282.9**^C^
		**362.3**^**B**^	**290.8**^F^	**324.0**^D^	**325.7**^B^
		**409.4**^**A**^	**322.3**^D^	**353.5**^C^	**361.7**^A^
Mean		**311.6**^**A**^	**254.3**^C^	**276.7**^B^	

Means followed by the same letter (s) within each row, column or interaction are not significantly different at 5% level. Significant values are in bold.

Concerning the effect of hydrogel, data in Table 5 indicated that hydrogel application positively affected crop fresh weight in both seasons. Fresh weight significantly increased with increasing hydrogel application rate. The highest value of fresh weight (g/plant) was 361.74 g followed by 325.69, 282.92 g for H_3_, H_2_ and H_1_, respectively. While the lowest value was 153.06 g under the control treatment H_0_. Regarding the effect of soil type on fresh weight of lettuce, the data showed that clay soil had significantly higher fresh weight than loamy sand and sand clay soil.

Moreover, with regard to the effect of interactions, the data revealed that the highest values of fresh weight were obtained by I_1_H_3_S_1_ (irrigation at 100% of ETc, hydrogel application rate of 0.3 g/kg soil and clay soil), followed by I_2_H_3_S_1_ and I_2_H_2_S_1_, respectively. The difference in fresh weight between I_1_H_3_S_1_ and I_2_H_3_S_1_ was not statistically significant. Same trend was observed in sandy soil, where the decrease in fresh weight was not statistically significant between I_1_H_3_S_3_ and I_2_H_3_S_3_. On the other hand, the lowest fresh weight was obtained under I_4_H_0_S_2_ (Deficit irrigation at 60% ETc, without hydrogel application in sandy soil).

Similarly, dry weight and number of leaves increased with increasing hydrogel application rates for all soil types, Table 6. These parameters were higher with higher irrigation levels and in plants grown in clay soil, followed sandy clay and sandy soil, respectively.

**Table 6:** Effect of hydrogel application rate on lettuce growth parameters in different soil types.

Soil type	S_1_				S_2_				S_3_			
Hydrogel level	H_0_	H_1_	H_2_	H_3_	H_0_	H_1_	H_2_	H_3_	H_0_	H_1_	H_2_	H_3_
No. leaves	26.3^G^	28.3^F^	38.8^B^	41.5^A^	26.3^G^	28.5^F^	27.4^FG^	32.8^D^	15.3^H^	31^E^	36.5^C^	33.3^D^
Dry weight	40.5^D^	44^A^	44.1^A^	44.3^A^	38.9^E^	40.1^D^	40.8^D^	40.7^D^	39.1^E^	42^C^	43.2^B^	42.7^BC^
Chlorophyl	41.2^D^	43.1^B^	43.7^B^	45.1^A^	35.3^G^	38.6^F^	38.1^F^	40.2^E^	39.3^F^	40.1^DE^	42.2^C^	42.9^BC^

Means followed by the same letter (s) within each row, column or interaction are not significantly different at 5% level.

### Effect of different treatments on water use efficiency

**Figure 2:**
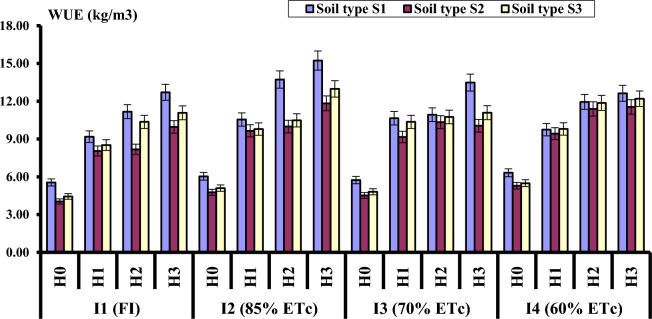
Effect of irrigation level, hydrogel rate and soil type on water use efficiency (kg/m^3^).

Data in Fig. 2 reveal that irrigation levels significantly affected water use efficiency (WUE). The highest WUE value was 10.01 (kg m^−3^) and was achieved under I_2_ (85% ETc), while the lowest WUE (8.61 kg m^−3^) was observed in fully irrigated plants (100% ETc). Concerning the effect of hydrogel application rate on WUE, data also showed that WUE was significantly affected by the hydrogel addition rate. The higher the hydrogel application rate, the greater WUE. Moreover, water use efficiency increased under H_3_ compared to H_0_ (untreated soil), from 5.91 to 13.52 kg m^−3^, from 4.65 to 10.85 kg m^−3^ and from 4.97 to 11.84 kg m^−3^ for clay, loamy sand and sandy clay soil respectively.

The interaction effect of different levels of irrigation, hydrogel application and soil type showed statistically significant variation for WUE. The highest WUE was recorded from I_2_H_3_S_1_ (15.23 kg m^−3^) followed by I_2_H_2_S_1_ (13.7 kg m^−3^) which was statistically similar to I_3_H_3_S_1_ (13.5 kg m^−3^). The lowest WUE was recorded from I_1_H_0_S_2_ (4.03 kg m^−3^). Given that I_2_H_3_S_1_ treatment resulted in the highest WUE and did not result in significant reduction in marketable yield (fresh weights), it can be adopted as irrigation water saving strategy. These results are consistent with who stated that hydrogels can enhance plant growth, plant productivity, and water use efficiency in arid and semiarid regions^28,29^.

### Effect of different treatments on soil hydro-physical properties

The effect of hydrogel rates on bulk density ρ (g cm^−3^) and hydraulic conductivity k (cm h^−1^) of different soil types is presented in Table 7. The data showed that there was no significant interaction between irrigation level and hydrogel addition rate on soil bulk density and hydraulic conductivity. A variable response to hydrogel rates was detected depending on soil type.

**Table 7:** Effect of hydrogel on soil properties.

	Soil type				Soil type			
	S_1_	S_2_	S_3_	Mean	S_1_	S_2_	S_3_	Mean
Hydrogel level	Density, ρ (g cm^−3^)				Hydraulic conductivity k_s_ (cm h^−1^)			
H_0_	1.19^E^	1.68^A^	1.31^D^	1.39^A^	0.42^F^	2.67^A^	1.24^D^	1.44^A^
H_1_	1.16^E^	1.62^A^	1.32^D^	1.36^AB^	0.40^FG^	2.61^B^	1.23^D^	1.41^B^
H_2_	1.18^E^	1.54^B^	1.29^D^	1.34^B^	0.41^F^	2.58^BC^	1.21^D^	1.40^B^
H_3_	1.08^F^	1.47^C^	1.28^D^	1.28^C^	0.38^G^	2.56^C^	1.16^E^	1.36^C^
Mean	1.15^C^	1.58^A^	1.30^B^		0.40^C^	2.60^A^	1.21^B^	

Means followed by the same letter (s) within each row, column or interaction are not significantly different at 5% level.

As for clay soil (S_1_), The lowest bulk density and hydraulic conductivity values were 1.08 g cm^−3^ and 0.38 cm h^−1^ and were achieved under H_3_, while the highest were 1.19 g cm^−3^ and 0.42 cm h^−1^ and were achieved under H_0_. There was no significant difference between H_1_ and H_2_ in both ρ and K_s_ values.

For loamy sand soil (S_2_), there was a significant decrease in density and hydraulic conductivity values at all hydrogel levels. The decrease of both ρ and K_s_ was proportional to the increase in hydrogel application rate. The lowest bulk density and hydraulic conductivity values were 1.47 g cm^−3^ and 2.56 cm h^−1^ and were achieved under H_3_, while the highest were 1.68 g cm^−3^ and 2.67 cm h^−1^ and were achieved under H_0_. For sandy clay soil (S_3_), K_s_ values decreased with increasing hydrogel, but the decrease was only significant at H_3_ application rate. Similar tendency was observed for ρ, where the lowest values were achieved under high rates of hydrogel (Table 7).

## Discussion

The results of this study showed that lettuce subjected to water stress had significantly lower yield and growth attributes than those with full irrigation requirements. These results are consistent with Kurunc^30^ and Ibrahim et al.^31^ who stated that water stress in lettuce is often accompanied by significant yield reductions and poor marketing quality. The sensitivity of lettuce to water stress may be attributed to its shallow root system that limits its ability to absorb water from deeper soil layers under water deficit conditions^21,32,33^. However, the application of super absorbent hydrogels mitigated these negative effects in water-stressed plants. The results revealed that hydrogel application enhanced fresh and dry weights, chlorophyll content and number of leaves. Despite the fact that lettuce plants are sensitive to water stress, hydrogel can be used to reduce the impact of water shortage (85% of ETc) without causing negative effects on production.

With respect to chlorophyll content, several studies have reported that chlorophyll can be used as a stress indicator^32,34^. Under water stress conditions, plants adapt to drought by stomatal closure to prevent water loss through transpiration. Stomatal closure results in degradation of photosynthetic pigments and the damage of chlorophyll structure under water deficits^35,36^. For all soil types, chlorophyl content significantly increased at higher hydrogel application rates, Table 6. Hence, it can be concluded that hydrogel can positively reduce plant water stress, enhances water availability and mitigates the negative effect of water deficiency on chlorophyll synthesis. These results are consistent with^32,37^, who reported significant decrease in chlorophyll content under water stress conditions and untreated soil.

Results also revealed that clay soil had significantly higher crop productivity than loamy sand and sand clay soil. This may be attributed to small and negatively charged particles of clay soils compared to sandy and sandy loam soils. In addition, clay soil often has a higher organic matter content leading to improved soil structure, water retention, and nutrient availability, all of which contribute to better crop growth and productivity^38,39^. In contrast, sandy soils are characterized by large pore spaces and large particles with less surface area for water absorption, leading to lower water retention and reduced plant available water^27,40^. Results revealed that hydrogel incorporation into sandy soil has improved the productivity and growth characteristics of the lettuce crop under DI conditions. The same trend of hydrogel for enhancing lettuce production under DI was also observed in clay soil as well. No significant difference in lettuce productivity under 85%ETc (I2) at hydrogel application rate of 0.3% for sandy and clay soils, Table 5. These findings are in line with Albalasmeh et al.^41^ and Sepehri et al.^42^, who reported that the crop productivity increased with increasing hydrogel concentration. Higher concentration of hydrogel can improve soil physical properties, enhance WUE and crop growth under DI conditions.

Based on lettuce yield and irrigation water amount, DI with 85% ETc (I_2_) was determined to be the best irrigation level in terms of WUE. According to Wallace^43^ and Howell^44^, WUE is the ratio of yield to plant water consumption and can be increased by maintaining the same yield while reducing irrigation water consumption. WUE was highest under I2 due to reduced amount of irrigation (the numerator in WUE equation), without significant yield reductions. Full irrigation may not achieve the highest WUE. Maximizing yield was the main objective of research studies in the twentieth century; recently the emphasis has changed to sustainable use of limited water resources. In areas experiencing water scarcity, the economic returns of water will be maximized by reducing the crop water consumption and increasing the irrigated area^7,45,46^.

Saturated hydraulic conductivity describes soil’s ability to transmit water when all pores are fully saturated. It provides information on the rate of water movement within the soil system, indicating possible water and nutrient leaching rates, which may affect plant growth and development and may lead to groundwater contamination^27,47^. The decrease in hydraulic conductivity values can be attributed to the hydrophilic properties of hydrogels. They swell by absorbing large volumes of water and decrease the drainage pore space between soil particles. As a result, more water is stored in the hydrogel structure and less water percolates from one soil layer to another. The results are also in agreement with^48,49^ who stated that K_s_ decreased with the increase in hydrogel application rate. When hydrogel concentration increased, the swelling of the hydrogel reduced the available paths for downward water movement. K_s_ values decrease considerably as soil becomes unsaturated since less pore space is filled with water^49,50^.

Regarding the effect of hydrogel on soil bulk density, the reduction in ρ values may be attributed to the displacement and rearrangement of soil particles around the swollen hydrogel particles. The hydrogel particles within the soil matrix absorb water and become larger in size. The soil volume increases and therefore the ratio of the dry soil mass to its volume decreases^49,51^. The obtained results are also in line with^49^ who reported that ρ values of fine textured soil was less affected by hydrogel application than the coarse sandy soil. They also stated that the minimum ρ value was found at the highest hydrogel rate (1.45 g cm^−3^), whereas the largest measured bulk density was found under the control (1.56 g cm^−3^). Soil bulk density (ρ) is a good indicator of soil compaction, the higher the bulk density the more compact it is. High ρ values tend to negatively influence soil aeration, and root growth. Hence, it can be concluded that hydrogels can decrease soil bulk density, improve soil porosity and enhance soil aeration.

## Conclusion

For different soil textures, the incorporation of hydrogel under deficit irrigation has significantly enhanced plant fresh and dry weight, chlorophyll content, and number of leaves. These results indicate sustainable lettuce performance under DI when using hydrogels. The difference in fresh weight in clay soil between I_1_H_3_S_1_ and I_2_H_3_S_1_ was not statistically significant. The same trend was observed in sandy soil, where the decrease in fresh weight was not statistically significant between I_1_H_3_S_3_ and I_2_H_3_S_3_. This means that despite the fact that lettuce plants are sensitive to water stress, hydrogel can be used to reduce the impact of water shortage (85% of ETc) without causing negative effects on production. On the other hand, the lowest fresh weight was obtained under I_4_H_0_S_2_ (Deficit irrigation at 60% ETc, without hydrogel application in sandy soil). Reduction in saturated hydraulic conductivity of soils confirms that this hydrophilic hydrogel can swell by absorbing large volumes of water and consequently improve water retention in sandy soils.

## Data Availability

The datasets used and/or analyzed during the current study available from the corresponding author on reasonable request.
